# Audiological and genetics studies in high-risk infants

**DOI:** 10.1590/S1808-86942011000600016

**Published:** 2015-10-19

**Authors:** Maria Francisca Colella-Santos, Maria de Fátima de Campos Françozo, Christiane Marques do Couto, Maria Cecilia Marconi Pinheiro Lima, Tatiana Guilhermino Tazinazzio, Arthur Menino Castilho, Edi Lucia Sartorato

**Affiliations:** 1 Doctoral degree, assistant professor and coordinator of the Speech Therapy Course, Medical School, UNICAMP; 2 Doctoral degree, assistant professor of the Speech Therapy Course, Medical School, UNICAMP; 3 Doctoral degree, assistant professor of the Speech Therapy Course, Medical School, UNICAMP; 4 Doctoral degree, assistant professor of the Speech Therapy Course, Medical School, UNICAMP; 5 Master's degree, speech therapist of the Neonatology Unit, Prof. Dr. José Aristodemo Pinotti Women's Hospital (CAISM); 6 Doctoral degree, otorhinolaryngologist of the Ophthalmology/Otorhinolaryngology Department, Medical School, UNICAMP; 7 Doctoral degree, researcher of the Genetics and Molecular Biology Center (Centro de Biologia Molecular e Genética), UNICAMP

**Keywords:** child, hearing, hearing loss, hearing tests

## Abstract

**Abstract:**

Hearing is one of the main ways with which one person can contact the external world; it plays a key role in their integration with society.

**Aim:**

The objective of this study was to analyze the results of the hearing, medical and genetic evaluation of high-risk infants who failed the newborn hearing screening.

**Materials and Methods:**

Clinical and experimental study. We assessed thirty-eight neonates, with ages between one and six months. The infants underwent the following procedures: medical interview; immittance testing; Brainstem Auditory Evoked Potential; Transient Evoked Otoacoustic Emission and otorhinolaryngological evaluation. DNA extraction from the oral mucosa was performed for genetic studies using the protocol method adapted from the Human Genetics Lab of the CBMEG/UNICAMP.

**Results:**

Regarding gender and presence of risk factors, significant statistically differences were not found in normal hearing infants and in those with hearing loss. Concerning gestational age, term infants were more affected by hearing loss. Hearing loss was identified in 58% of the sample, conduction hearing loss represented 31.5% (12/38) and neurossensory 28.9% of cases. There were none of the genetic mutations most commonly seen in cases with a genetic etiology.

**Conclusion:**

Hearing loss was identified in the majority of High-risk infants.

## INTRODUCTION

Hearing is one of the main forms by which individuals establish contact with the external world, and is thus of utmost importance for integration within society.

The estimated incidence of significant and bilateral hearing loss is one to three for each thousand newborns at low risk for hearing loss^1^,^2^; these numbers increase to about two to five for each one hundred newborns in intensive care units (ICUs)^1^,^3^,^4^, or 10.2% as reported by Lima et al.^5^,^6^ According to the Brazilian Geography and Statistical Institute (IBGE) the incidence of hearing loss in Brazilians in the year 2000 census was 16.7%; in the state of Sao Paulo, this rate was 16.4%^7^. Hearing loss is the most common congenital abnormality; it is more prevalent than other routinely screened diseases. Hearing loss is 100 times more prevalent than phenylketonuria and 10 times more prevalent than hypothyroidism^8^. Infant hearing loss is considered a serious public health issue because of its high prevalence and the ensuing consequences, which include the infant's language, cognitive, intellectual, cultural, and social development^9^,^10^.

Universal neonatal hearing screening has been recommended as the main strategy to reduce the age at which a diagnosis of hearing loss may be made^11^,^12^. This is the first step in a neonatal hearing health program, and should be part of a multidisciplinary approach to diagnosis involving mainly speech therapists, otorhinolaryngologists, and even geneticists at specific centers. After a diagnosis, interventions such as sound amplification and rehabilitation should be started. An early diagnosis followed by medical and phonoaudiological interventions may help infants to establish contact with the world of sound at the time of central nervous system plasticity in the first year of life, which will increase nerve connections and allow for improved results in auditory rehabilitation and general development of infants with hearing loss^13^,^14^.

A test battery with behavioral and electrophysiologic tests is recommended for the diagnosis of hearing loss, such as: observing auditory behavior relative to calibrated and non calibrated sounds, acoustic immittance testing, otoacoustic emissions, and brainstem auditory evoked potential (BAEP). These suggested tests help define the type, grade, and configuration of hearing loss, which result in a differential diagnosis, interventions, and the indication of hearing aids for sensorineural, mixed, or conductive hearing loss^15^. It is important that the etiology of deafness be established to define the prognosis and implement appropriate measures.

There are several causes of congenital hearing loss – those acquired during the prenatal period or within the first days after delivery. The causes of hearing loss may be genetic or environmental. The etiology of genetic origin hearing loss may be syndromic, those associated with craniofacial or neck malformations, skeletal dysplasia, skin or ocular abnormalities, neurologic diseases, renal or metabolic dysfunction, and others. It has been estimated that 30% of cases of prelingual deafness are syndromic and 70% are non-syndromic^16^. Non-syndromic deafness may have several patterns of inheritance: linked to the X chromosome (DFN) in 1-3% of cases; autosomal dominant forms (DFNA) in 15% of cases; and autosomal recessive forms (DFNB) in 80% of cases. Furthermore, there are cases of maternal inheritance due to mitochondrial gene mutations^16^. In developed countries, about 60% of hearing loss cases are genetic in origin^17^.

Environmental factors include congenital infections, perinatal, and postnatal factors.

Thus, the purpose of this study was to investigate the results of an audiologic, otorhinolaryngologic, and genetic evaluation of high-risk infants that failed in neonatal hearing screening, taking into account the variables male/female sex, number of risk indicators, and gestational age. Hearing loss was also analyzed according to type, grade, affected side, and possible etiology.

## MATERIALS AND METHODS

A cross-sectional contemporary cohort study was carried out. It was approved by the institutional review board of the Research Ethics Committee of the School of Medical Sciences of the University of Campinas (FCM/ UNICAMP). The reference number was 028/2008.

All infants born at the Women's Hospital - Prof. Dr. José Aristodemo Pinotti – CAISM/UNICAMP that remained in the neonatal ICU and that failed hearing screening, from February 2009 to March 2010 were enrolled. Infants born in other hospitals or that did not undertake all the evaluations within the study period were excluded. There were 52 infants sent for a diagnostic assessment; this number is close to the sample size based on the mean monthly delivery number at the hospital (250 deliveries) and the number of newborns that are admitted into the ICU (40 per month). Thus, there were 500 newborns in 12 months, of which on average 10% (50 infants) failed in hearing screening^6^. The hearing screening test was the automatic brainstem auditory evoked potential (A-BAEP). An Algo 2e color NATUS test equipment was used in an acoustic booth. Infants passed the hearing screening test when the response was present for 35 dB bilaterally on the A-BAEP.

A staff speech therapist of the hospital Audiology Diagnostic Laboratory carried out the audiological evaluation. The infants were aged 1 to 6 months when tested; the procedures were as follows: a clinical history, evaluation of the middle ear status, BAEP testing (electrophysiologic threshold and auditory pathway integrity), and transient otoacoustic emissions (TOAE). Infants slept naturally while the procedures were applied.

The clinical history was taken from family members to gather the following data: identification, the hospital discharge report, and information about motor, auditory and language development. The JCIH^18^ risk factors were used as indicators of risk; these were also gathered from the neonatologist's hospital discharge report.

The electrophysiologic threshold and auditory pathway status were assessed by BAEP using an Eclipse EP 25 Interacoustics device with in-ear phones. Auditory pathway status was investigated with non-variable 80 dB clicks presented 19 times per second, which make it possible to evaluate auditory pathways and identify issues up to the brainstem. The electrophysiologic threshold was gathered by sloping stimuli until reaching the lowest stimulus intensity that caused the V wave to appear. The stimulus was applied twice to obtain a reproducible tracing and make sure the response was present. Surface electrodes were attached over the right and left mastoid and the frontoparietal regions of infants after cleaning the skin with abrasive paste and applying electrolytic paste. The following parameters were assessed: presence of waves I, III, and V; absolute wave I, III, and V latencies; I-V, I-III, and III-V interpeak latencies; wave V amplitude relative to wave I amplitude; and I-V interpeak or wave V latency interaural difference. TOAE were measured by an ILO 292 USBII device.

The tympanometric curve was used to evaluate the middle ear; the probe tone was set at 1,000 Hz and the ipsilateral acoustic reflex was measured from 500 to 4,000 Hz. A 235 H, Interacoustics device was used.

Responses in each test were recorded on answer sheets. Normal hearing was considered as a click electrophysiologic threshold under 30 dB, absolute and interpeak latencies within expected values for the gestational age^19^, the presence of TOAE^20^, a type A tympanometric curve, and the presence of an ipsilateral acoustic reflex^21^,^22^.

Infants with abnormal results in at least one auditory test were sent to an otorhinolaryngologist to be evaluated. One of the researchers supervised this assessment, which consisted of otoscopy to examine the outer ear canal and the tympanic membrane, and imaging if needed.

Hearing was classified as normal or impaired depending on the analysis of the audiologic and otorhinolaryngologic assessments. Hearing loss was classified according to the type^23^, the grade (Silman & Silverman^24^), and whether unilateral or bilateral. Genetic screening was done by DNA extraction from oral mucosa sampled by the examiner after performing auditory testing in infants that failed hearing screening. The 35delG mutation was investigated in the DNA sample by using standardized AS-PCR at the Human Molecular Genetics Lab - CBMEG. (patent no. P10005340-6; test method for deafness of genetic origin). The PCR was used to search for the D(*GJB6*-D13S1830) and D(*GJB6*-*D13S1854*) deletions with previously reported primers^25^. A single diagnostic test involving both deletions in the same PCR was done. Mitochondrial mutations were analyzed by amplifying DNAmt fragments of the *MTRNR1* gene to detect the A1555G mutation; the abovementioned primer pairs were used. Restriction analysis to detect mutations was applied to amplification products.

The statistical analysis was made with the SAS software version 9.1.3. The significance level was 5%, and was indicated by an asterisk (*).

## RESULTS

There were 52 infants that failed hearing screening and that were referred for diagnosis. Of these, 38 infants (73%) participated in the study and underwent the full diagnostic process. The remaining infants either did not come to scheduled visits or failed to sleep after BAEP testing, among other factors, thereby not undergoing the full diagnostic procedure within the study period.

Table 1 shows the results for infants with normal or impaired hearing, related with the variables male/ female sex, indicators in the clinical history, and gestational age.

**Table 1:** Nursing infants that failed hearing screening, according to normal or impaired hearing and the variables sex, gestational age, and number of risk indicators.

	Normal hearing		Hearing loss		*p*-value^a^
	n	(%)	n	(%)	
SEX					
F	3	18.8	10	45.5	
M	13	81.3	12	54.6	0.867
No. of RI					
1	1	6.3	0	0	
2	8	50	13	59.1	
3	2	12.5	2	9.1	
3 or +	5	31.3	7	31.8	0.7403
GA					
PTN	11	68.8	8	36.4	
TN	5	31.3	14	63.6	0.0487*

RI: Risk indicators; GA: Gestational age.

PTN: Preterm newborn; TN: term newborn.

a Fisher's exact test / chi-square test.

Table 2 shows infants distributed according to the audiologic and otorhinolaryngologic diagnosis.

**Table 2:** Nursing infants that failed hearing screening, according to the audiologic and otorhinolaryngologic diagnosis.

Diagnosis		Total			
		N	%	N	%
Normal hearing		16	42.2	16	42.2
Conductive loss	Unilateral	2	5.2		
	Bilateral	10	26.3	12	31.5
Sensorineural loss	Unilateral	1	2,6		
	Bilateral	9	23.7	10	26.3
Total		38	100	38	100

The 35delG mutation on the conexin 26 (*GJB2*) gene was not encountered, neither were the D(*GJB6-*D13S1830) and Δ(*GJB6-*D13S1854) deletions on the *GJB6* gene and the A1555G mutation on the *MTRNR* mitochondrial gene.

Table 3 presents infants with sensorineural hearing loss, considering the grade and etiology.

**Table 3:** Nursing infants according to the grade and etiology of sensorineural hearing loss.

	Environmental causes	Genetic causes	Total
Moderate	1	0	1
Severe	4	0	4
Profound	5	0	5
Total	10	0	10

## DISCUSSION

Neonatal hearing screening is the main method to detect early hearing loss. The procedure needs to be fast, simple, and able to select individuals with the highest probability of abnormal function^26^. Newborns in an neonatal ICU of the Caism/UNICAMP underwent hearing screening preferably before being discharged from the hospital; the procedures were automatic. A-BAEP has been recommended for use mainly in neonates in ICUs because of a higher incidence in this population of risk indicators for retrocochlear abnormalities involving inner hair cells, auditory pathways and/or the brainstem^18^,^27^.

Thirty-eight neonates failed the hearing screening, of which 25 (66%) were male and 50% were premature. Most of the children were male, which reflects the demography of most neonatal ICUs^28^.

All infants had at least one risk indicator in the clinical history (Table 1).

There was no statistically significant difference in normal and impaired hearing neonates in relation to the variables gender and number of risk indicators. On the other hand, term neonates were more affected by hearing loss than premature infants, and this finding was statistically significant (Table 1). Term neonates in this study required intensive care because of severe intercurrences at birth such as anoxia, congenital malformation, syndromes, and congenital infection – all of which are risk indicators for hearing loss. Published studies describe neonatal features found in infants with hearing loss diagnosed by neonatal hearing screening, which includes gestational age over 37 weeks and birth at weight over 2,500 grams^28^.

Analysis of the tests showed that 42% (16/38) of children had normal results in all tests (Table 2). Their failure in hearing screening and normal auditory test results suggests either a temporary conduction type change in the auditory system that regressed, or delayed myelination of auditory pathways to the brainstem. Infants with risk indicators in their clinical histories that might indicate progressive and/or late onset hearing loss were referred for monitoring of hearing and language development.

Mostly bilateral conductive hearing loss was present in 31.5% (12/38) of the sample (Table 2). Conductive hearing loss was present in 55% (12/22) of children. Audiological testing (BAEP) revealed absence of TOAE, type B tympanometric curve, absence of ipsilateral acoustic reflexes, and a normal auditory pathway at 80 dB. We found that the Down syndrome was present in 4 of 12 (33.33%) cases of conductive hearing loss; the external ear and the outer ear canal were malformed in one of the cases of unilateral conductive hearing loss.

Bone et al.^29^ published similar results showing conductive abnormalities in children that failed hearing screening; the most common cause in these cases was otitis media. These abnormalities were variable, episodic, ranging from mild to moderate, never beyond 50 dB^30^. Otitis media is highly prevalent in infancy, especially in high risk infants; middle ear secretions occur mostly between the 4^th^ and 12^th^ months, and occur least in the first three months of life^30^, ^31^, ^32^, ^33^. It is multifactorial and associated with lack or early interruption of breastfeeding, feeding in decubitus, a first episode of acute otitis media before six months of age, immature or deficient immune system, stays in nurseries where common colds and the flu occur frequently, and passive smoking^34^. Infants presenting secretory otitis during the neonatal period are at a highest risk for chronic otitis media within the first year of life^35^. Studies on the occurrence and recurrence of middle ear secretion have shown a higher frequency of recurrences of four or more episodes in males; the highest incidence of middle ear secretion was in the first month of life. The authors also found that infants breastfed until 6 months of age had a higher recurrence rate – four or more episodes. The opposite was seen in infants nursed during more than 10 months. The authors recommended programs to prevent, diagnose, and treat otitis, especially because the first years of life are critical for development; they also suggested that healthcare professionals encourage breast feeding^30^.

Preventive measures, such as encouraging breast feeding, positioning the infants adequately while nursing, avoiding passive smoking, and other initiatives, may minimize conductive hearing losses. Such losses impair the development of hearing and language by causing sensory deprivation fluctuating hearing that is typical of otitis media.

The incidence of hearing loss in Down syndrome infants ranges from 2.6% to 67.5%; it has been attributed mainly to a high rate of secretory otitis media^36^,^37^. Strome et al.^37^ reported 70% middle ear effusion in 107 Down syndrome patients aged below 1 year. Several factors appear to increase the incidence of secretory otitis media, including: abnormal auditory tube anatomy, abnormal ossicular chain, dysfunction of muscles that open the auditory tube, and stenosis of the external acoustic meatus^37^.

These infants were receiving otorhinolaryngological treatment, periodic audiologic evaluations, and were monitored for language and hearing development. Published reports have shown that some cases of middle ear involvement resolve spontaneously without harm to development, whereas in other cases, the disease becomes chronic. In this case it may compromise language and educational development, and if left untreated, may progress and involve the mastoid cells or even the cranial cavity, which leads to serious complications including inner ear involvement and sensorineural hearing loss^29^,^33^.

We found sensorineural hearing loss in 28.9% (10/38) of the sample; five cases were moderate to severe, and five cases were profound loss (Table 3). These infants were referred for evaluation and fitting of hearing aids, guidance for parents, and phonoaudiological rehabilitation. The most frequent risk indicators in the clinical histories were elevated blood bilirubin levels, perinatal anoxia and mechanical ventilation, and ICU stay for more than five days.

The results of three infants among the cases of bilateral profound sensorineural hearing loss were compatible with the auditory neuropathy spectrum disorder (ANSD), which consist of cochlear microphonism, absence of waves I, III, and V at 100 dB in BAEP testing, TOAE, a type A tympanometric curve, and absence of acoustic reflexes. An elevated bilirubin level was a risk indicator in the three cases – bilirubin levels were above 28 mg/dL.

Findings in the ANSD include 8^th^ cranial nerve and/or brainstem dyssynchrony, disordered inner hair cells, dysfunctional spiral ganglion fibers^38^, abnormal synaptic afferent transmission between inner hair cells and the 8^th^ cranial nerve^39^, and normally functioning outer hair cells. Most cases present bilateral abnormalities ranging from severe to profound loss. However, unilateral cases and moderate losses have been reported, which suggests an inhomogeneous entity^40^. The ANSD may occur in the absence of any other apparent medical condition; a history of elevated blood bilirubin levels in the perinatal period and asphyxia or anoxia is frequent^40^. The ANSD is much more common in infants admitted into neonatal ICUs^40^. The reported prevalence in the literature ranges from 0.2% to 4% in infants at risk for hearing loss, and 0.5% to 15% in infants with known hearing loss^40^,^41^. The prevalence is lower in studies that enroll older children or those that study in schools for the deaf; it is possible that the ANSD is only diagnosed objectively in the first months of life when otoacoustic emissions are present because of normal outer hair cell function. As the disease progresses, these cells become inactive or may have been injured by hearing aids in undiagnosed infants. Declau et al.^17^ reported a diagnosis of ANSD in two cases (4.2%), one of which had elevated blood bilirubin levels in the perinatal period and the other had no risk indicators. It is important to make the differential diagnosis of sensorineural hearing loss because approaches will vary compared with measures taken to treat other forms of permanent hearing loss.

All study subjects were normal for the 35delG conexin gene 26 *(GJB2*) mutation. The V27I polymorphism on the *GJB2* gene was found in subject 1 in heterozygosis, which is not relevant for hearing loss, as is the case for silent mutations. Although the 5delG mutation was not found in the sample after screening, it is extremely important since it is present in 70% of cases of deafness when the *GJB2* gene is involved. The prevalence of carriers of the 35delG mutation in Brazil, as encountered in a survey of 620 neonates in Campinas (Sao Paulo state) is 0.97% – about 1:103 heterozygotes^42^. A negative result for mutations in the *GJB2* gene reduces the empirical risk of a genetic cause of deafness. Besides the *GJB2* gene, Δ*(GJB6-D13S1830)* and Δ*(GJB6-D13S1854)* deletions on the *GJB6* gene were also analyzed. None of our subjects had any of these deletions. We also tested for the A1555G mitochondrial mutation, which is associated with hearing loss and use of aminoglycoside antibiotics, and did not find this abnormality in any of our study subjects.

Declau et al.^17^ undertook a prospective study of audiological findings and causes of hearing loss in 170 infants that failed neonatal hearing screening, of which 13 were admitted to the neonatal ICU. Infants that failed were referred for electrophysiologic testing (BAEP, stable state responses and/or behavioral tests). Permanent hearing loss was present in 61.5% of infants admitted to a neonatal ICU. The male to female ratio of hearing loss was 3/0. The mean hearing loss was 60 dBHL. The most prevalent risk factors were mechanical ventilation, low weight, and elevated blood bilirubin levels. Environmental causes were the etiology in 39.6% of hearing loss cases; the most frequent was congenital cytomegalovirus infection (18.8% of cases).

According to the literature, the cause of hearing loss is genetic in 50% of cases and environmental in another 50% of cases in developed countries^42^,^43^. Also, the most important environmental factors for hearing loss are congenital infections, ototoxicity, prematurity, and neonatal anoxia^43^.

In our study, environmental factors in the history of infants were the most likely causes, because no genetic mutations commonly implicated in hearing loss were found (Table 3). Identifying the etiology of hearing loss is a relevant point that provides new information for auditory rehabilitation, the prognosis, and the family. Furthermore, these studies may help clarify the epidemiological factors of hearing loss, which may support preventive and surveillance programs.

## CONCLUSION

Analysis of audiologic, otorhinolaryngological, and genetic findings in high risk nursing infants that failed hearing screening showed that the frequency of hearing loss was higher in term neonates compared to premature neonates. Children with predominantly bilateral conductive and sensorineural hearing loss had a similar distribution. The likely causes of sensorineural hearing loss are environmental factors. The diagnosis and etiology of deafness are extremely important for establishing the prognosis and appropriate therapy.
